# Facial appearance associates with longitudinal multi-organ failure: an ICU cohort study

**DOI:** 10.1186/s13054-024-04891-6

**Published:** 2024-04-02

**Authors:** Eline G. M. Cox, Bas C. T. van Bussel, Nerea Campillo Llamazares, Jan-Willem E. M. Sels, Marisa Onrust, Iwan C. C. van der Horst, Jacqueline Koeze, SICS Study Group, Geert Koster, Jacqueline Koeze, Renske Wiersema, Frederik Keus, Iwan C. C. van der Horst, Willem Dieperink, Marisa Onrust, Nynke van der Veen, Alexander Irk, Arlinde Roelofs, Leonie Tijsma, Eline Cox, Nerea Campillo Llamazares, Lesley Holzhauer

**Affiliations:** 1 Department of Critical Care, University of Groningen, University Medical Center Groningen, Groningen, The Netherlands; 2 Department of Intensive Care Medicine, Maastricht University Medical Center+, University Maastricht, Maastricht, the Netherlands; 3 Cardiovascular Research Institute Maastricht (CARIM), Maastricht University, Maastricht, The Netherlands

**Keywords:** Gut feeling, Multi-organ failure, Facial appearance, Facial monitoring, Critical care

## Abstract

**Background:**

Facial appearance, whether consciously or subconsciously assessed, may affect clinical assessment and treatment strategies in the Intensive Care Unit (ICU). Nevertheless, the association between objective clinical measurement of facial appearance and multi-organ failure is currently unknown. The objective of this study was to examine whether facial appearance at admission is associated with longitudinal evaluation of multi-organ failure.

**Methods:**

This was a sub-study of the Simple Intensive Care Studies-II, a prospective observational cohort study. All adult patients acutely admitted to the ICU between March 26, 2019, and July 10, 2019, were included. Facial appearance was assessed within three hours of ICU admission using predefined pictograms. The SOFA score was serially measured each day for the first seven days after ICU admission. The association between the extent of eye-opening and facial skin colour with longitudinal Sequential Organ Failure Assessment (SOFA) scores was investigated using generalized estimation equations.

**Results:**

SOFA scores were measured in 228 patients. Facial appearance scored by the extent of eye-opening was associated with a higher SOFA score at admission and follow-up (unadjusted 0.7 points per step (95%CI 0.5 to 0.9)). There was no association between facial skin colour and a worse SOFA score over time. However, patients with half-open or closed eyes along with flushed skin had a lower SOFA score than patients with a pale or normal facial skin colour (*P*-interaction < 0.1).

**Conclusions:**

The scoring of patients’ facial cues, primarily the extent of eye-opening and facial colour, provided valuable insights into the disease state and progression of the disease of critically ill patients. The utilization of advanced monitoring techniques that incorporate facial appearance holds promise for enhancing future intensive care support.

**Supplementary Information:**

The online version contains supplementary material available at 10.1186/s13054-024-04891-6.

## Introduction

Clinical examinations are an integral part of the comprehensive care of critically ill patients. Healthcare providers instinctively integrate a patient’s face and overall appearance into their bedside assessment. These visual cues can provide valuable information about the patient’s well-being, and allow the healthcare provider to make a more informed decision regarding patient treatment and overall care [1, 2].

Previous studies have shown that facial cues reflecting illness can be identified in patients [3, 4]. However, the association between facial appearance and disease severity during Intensive Care Unit (ICU) admission remains unclear. Understanding this association could provide valuable insights into the potential of facial monitoring related to a patient’s status in the ICU. In this proof-of-concept study, we investigated the extent of eye-opening and facial skin colour and their association with longitudinal multi-organ failure as measured by the maximum Sequential Organ Failure Assessment (SOFA) score in the first week of ICU admission.

## Methods

﻿This was a sub-study of the Simple Intensive Care Studies II (SICS-II), a single-center, prospective observational study (NCT03577405) [5]. All acutely admitted patients above 18 years of age with an expected ICU stay of at least 24 h were included between March 26, 2019, and July 10, 2019. Thirty bachelor medical and nursing students volunteered in shifts to support research in the ICU. After an acute admission, a student was called by phone and came to collect bedside data [5]. Within three hours after ICU admission facial appearance was assessed using predefined standardized pictograms depicting the patient’s face based on the extent of eye-opening (open, half-open, slit, closed) and facial skin colour (pale, normal, flushed) (Additional file 1: Figure S1A). When the patient’s eyes were fully closed, it was assessed whether they were mechanically ventilated. The pictograms were created based on a previous study, which found that a pale face and partially closed eyes indicated illness [2]. The faces were neutral to accommodate all skin types and ensure universal applicability (Additional file 1: Figure S1B). The observers were blinded to the clinical data as they had no access to the patient’s Electronic Health Record (EHR) system.

The SOFA score was assessed by a student-who was blinded from the facial assessment at admission and who did not perform clinical tasks-based on the data from the EHR system. The daily values from the morning report at 10:00 a.m. or as close to 10:00 a.m. as possible were selected (Additional file 1: Table S1). When a component or variable of the SOFA score was missing on a day the patient was in the ICU, it was completed based on available clinical information.

Continuous variables are presented as means (with standard deviations (SD)) or medians (with interquartile ranges (IQR)) depending on distributions and categorical data as proportions. Generalized estimation equations (GEE) were used to investigate the association between categories of either the extent of eye-opening and categories of facial skin colour and longitudinal SOFA scores. An exchangeable correlation structure was used. The model was adjusted for potential confounders; age, gender, BMI, and Acute Physiology and Chronic Health Evaluation (APACHE) IV score. Effect modification between facial skin colour and the extent of eye-opening and their association with longitudinal SOFA score was investigated using interaction term analysis. The GEE model included the following determinants: eyes half-open, slit, closed, and mechanically ventilated (4), normal and flushed facial skin colour (2), with their interaction terms (4 × 2). Eyes open and pale facial colour served as reference categories. Serial SOFA score was the outcome variable. Analyses were performed using Stata version 17 (StataCorp, College Station, TX, USA). We report β with 95% CI and *p*-values. *P*-values < 0.05 and *P*-interaction < 0.1 were considered statistically significant.

## Results

**Fig. 1:**
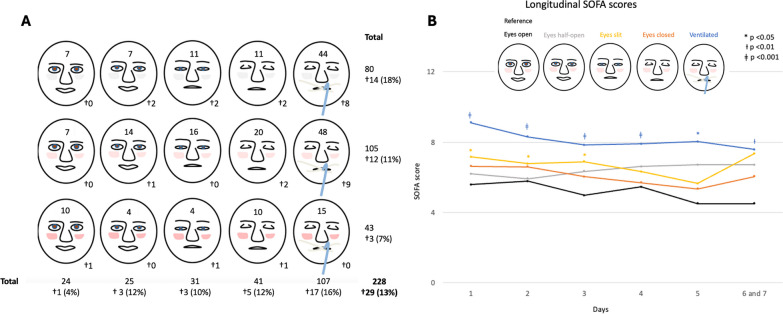
The predefined pictograms. **A** Distribution of the different pictograms. **B**. Longitudinal trajectories of SOFA scores over time according to eye-opening*. †died in-hospital; SOFA: Sequential Organ Failure Assessment;  *Data was collected until day seven of ICU admission because most deteriorations are expected to occur within that timeframe, and 84% of patients were discharged within seven days. Therefore, the SOFA scores on days 6 and 7 were grouped. SOFA scores were calculated only for the days the patient was admitted to the ICU

In total, 228 patients were included in the analyses (Additional file 1: Figure S2). The median time from ICU admission to inclusion was 1.8 h (IQR 1.1–2.7), the median age was 64 ([IQR] 52–72) years, and most patients were male (61%). Median ICU length of stay was 2.0 (IQR 1.0–4.5) days (Table 1). Availability of SOFA score data varied depending on ICU length of stay (Additional file 1: Table S1). The SOFA score was highest at 1.7 days (SD ± 1.0), with a mean of 6.0 points (SD ± 3.3). Almost half of the patients were mechanically ventilated (Fig. 1A).

**Table 1:** Baseline characteristics of the 228 patients included in this study

**Variable**	**Patients**	
Age, years, median (IQR)	64.0	(52.0–72.0)
Sex, male, *n* (%)	139	(61%)
BMI, kg/m^2^, median (IQR)	25.6	(23.0–29.0)
Reason for admission, n (%)		
Medical	153	(67%)
Acute surgery	74	(29%)
Medical History, n (%)		
Diabetes Mellitus	34	(15%)
Hypertension	77	(34%)
Myocardial Infarction	18	(8%)
SOFA score at admission, median (IQR)	4	(3–7)
APACHE score at admission, mean [SD]	70	28
Length of stay at ICU, days, median (IQR)	2.0	(1.0–4.5)
90-day mortality, n (%)	61	(27%)

*IQR* interquartile range, *BMI* body mass index, *SOFA* Sequential organ failure assessment

GEE analysis showed that the SOFA score was statistically significantly different between categories. For each step from open eyes to closed eyes and mechanically ventilated, the SOFA score was, on average, 0.7 points (95%CI 0.5 to 0.9) higher over time. This result was similar when adjusted for age, gender, BMI, and APACHE IV score (*β* (95%CI); 0.6 (0.4 to 0.8)). After categorization, compared to patients with open eyes, those with their eyes half-open (0.8 points; 95%CI − 0.3 to 1.8), slit (1.3 points; 95%CI 0.4 to 2.3) or closed (0.9 points; 95%CI − 0.02 to 1.8), or those who were mechanically ventilated (2.8 points; 95%CI 2.0 to 3.6; *p* < 0.001) had, on average, over seven days, a higher SOFA score (Fig. 1B). The SOFA score decreased in all groups over time, except for patients with their eyes half-open.

Patients who exhibited flushed skin were more likely to survive (Fig. 1A). After categorization, patients with pale or flushed skin had, on average, a similar longitudinal SOFA score compared to patients with a normal skin colour (0.1 point 95%CI − 0.6 to 0.7 and − 0.1 point 95%CI − 0.9 to 0.7, respectively). The association between the extent of eye-opening and longitudinal SOFA scores was modified by facial skin colour (*P*-interaction < 0.1) (Additional file 1: Figure S3). The impact of flushed skin on the SOFA score was present in individuals with half-open or closed eyes, whereas skin tone did not appear to modify the effect on the SOFA score with other eye categories.

## Discussion

These results support three conclusions: (1) the extent of eye-opening at ICU admission was associated with worse longitudinal SOFA scores; (2) the trait of facial skin colour itself was not associated with a worse SOFA score over time, and (3) importantly for the concept of facial monitoring techniques, facial skin colour interacted with the extent of eye-opening, modifying the association with longitudinal SOFA score. The last aligns with the observation of a lower mortality rate in patients with flushed skin and may suggest an adequate response to, e.g., infectious disease.

Prior research has established a connection between an individual's health and the characteristics of their facial appearance, suggesting that one can gauge a person's health status by examining their face [6–8]. One study demonstrated that persons in photographs rated as 'sick' more often had pale skin, pale lips, and partially closed eyes [2]. Another study showed that ward patients who had closed eyes, depressed lip corners, parted lips, and heads turned were more likely to deteriorate [4]. Moreover, verbal communication, body language, and facial appearance could provide valuable information about patient’s severity of injury when they enter the Emergency Department [3]. Our observations, in general, support the concept that clinical gestalt is more complex than the sum of its traits.

### Implications and generalizability

In the past decade, phenotypic decision support for rare disorders has been improved by developing innovative facial recognition tools, such as GestaltMatcher [9–11]. It is fascinating whether facial or overall appearance derived by intelligent monitoring sensors can aid in care for hospitalized patients by providing real-time information for predicting disease progression. Smart cameras or intelligent systems have the potential to decrease the monitoring workload for nurses, optimizing workforce efficiency and enabling them to concentrate more on providing care. However, further research is needed to explore this possibility.

### Strengths and limitations

Facial assessment of a large cohort of acutely admitted patients was conducted shortly after admission when disease severity was minimally impacted by interventions or treatment decisions.

Limitations include the fact that we measured only two traits of facial appearance. Our study is a single-center study, and we did not record inter-observer agreement. Further, although the pictograms were developed to be suitable for all skin types, it is crucial to consider that individual skin tones can still influence the reported colour of the cheeks. Estimating facial skin colour may pose a challenge with individuals having darker skin tones, which could lead to an underestimation of associations between facial skin colour and longitudinal SOFA scores in our study. However, this limitation underscores the importance of incorporating inclusivity in the evaluation of facial appearance through the integration of advanced technologies in future clinical research [12].

Also, ethnicity is not registered in Dutch hospitals; this limitation hinders the external validity of this study. In addition, the neurological component of the SOFA score is derived from the Glasgow Coma Scale, which assesses eye-opening. This evaluation involves stimulating the patients to open their eyes. The patient was only observed in our study; therefore, this possible influence may only apply to mechanically ventilated patients. This could have led to underestimation of the results.

## Conclusion

The scoring of patients’ facial cues, primarily the extent of eye-opening and facial colour, provided valuable insights into the disease state and progression of the disease of critically ill patients. A complete face provides more information than individual traits do. The utilization of advanced monitoring techniques that incorporate facial appearance holds promise for enhancing future intensive care support.

### Supplementary Information

**Additional file 1: Fig. S1**. A Standardized pictograms designed for this proof-of-concept study. B Simulation of the pictograms for different skin colours. **Fig. S2**. Flowchart of study inclusion. **Fig. S3**. Interaction between the two traits of gestalt; eye-opening and facial skin colour. SOFA sequential organ failure assessment; bars indicate standard error of the mean. **Table S1**. The SOFA score*. a: with respiratory support; b: adrenergic agents administered for at least one hour (doses given are in µg/kg × min). MAP mean arterial pressure, CNS central nervous system; SOFA sequential organ failure assessment. *After completing the data, every patient had a SOFA score for each day at the ICU, resulting in 852 serial SOFA scores. On day one of admission, all patients had a SOFA score; on day two, 214 (94%) patients had a SOFA score, and on day three, 139 (61%) patients, and on day four, 96 (42%) patients had a SOFA score. On day five, 72 (32%) patients, day six, 58 (25%), and day seven, 45 (20%) patients had a SOFA score.

## Data Availability

The datasets used and/or analyzed during the current study are available from the corresponding author upon reasonable request.

## Abbreviations

ICU: Intensive care unit
SOFA: Sequential organ failure assessment
SICS: Simple intensive care studies
NICE: National intensive care evaluation
IQR: Interquartile range
SD: Standard deviation
GEE: Generalized estimation equations
