# Supplementary material for: Facial appearance associates with longitudinal multi-organ failure: an ICU cohort study
Source: Crit Care. 2024 Apr 2;28:106. doi: 10.1186/s13054-024-04891-6 (PMC10988949; doi:10.1186/s13054-024-04891-6)
Supplement: Supplementary file 1 — Additional file 1: Fig. S1. A Standardized pictograms designed for this proof-of-concept study. B Simulation of the pictograms for different skin colours. Fig. S2. Flowchart of study inclusion. Fig. S3. Interaction between the two traits of gestalt; eye-opening and facial skin colour. SOFA sequential organ failure assessment; bars indicate standard error of the mean. Table S1. The SOFA score*. a: with respiratory support; b: adrenergic agents administered for at least one hour (doses given are in µg/kg × min). MAP mean arterial pressure, CNS central nervous system; SOFA sequential organ failure assessment. *After completing the data, every patient had a SOFA score for each day at the ICU, resulting in 852 serial SOFA scores. On day one of admission, all patients had a SOFA score; on day two, 214 (94%) patients had a SOFA score, and on day three, 139 (61%) patients, and on day four, 96 (42%) patients had a SOFA score. On day five, 72 (32%) patients, day six, 58 (25%), and day seven, 45 (20%) patients had a SOFA score. [file 13054_2024_4891_MOESM1_ESM.docx]

**SUPPLEMENTAL MATERIAL**

**Supplemental Figure 1.**

**A.** Standardized pictograms designed for this proof-of-concept study.

**B.** Simulation of the pictograms for different skin colours

**
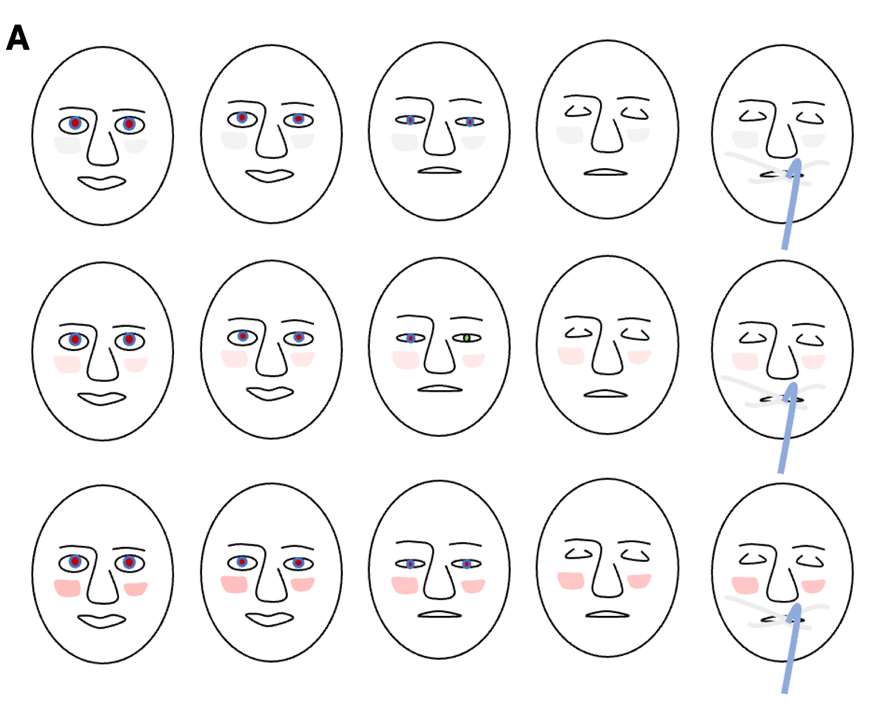
**


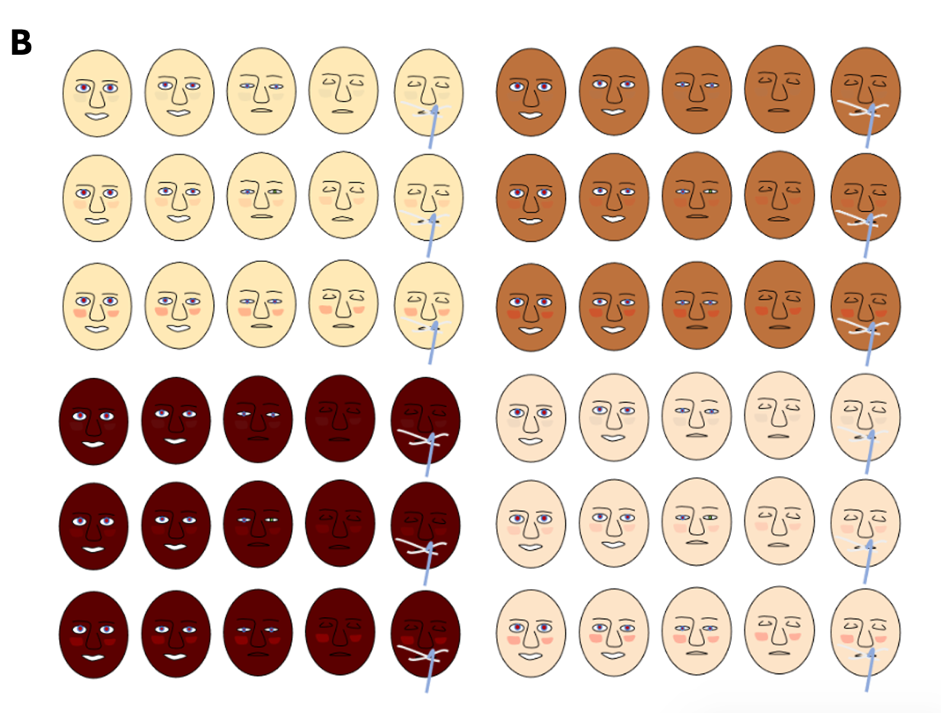


**Supplemental Figure 2**. Flowchart of study inclusion**
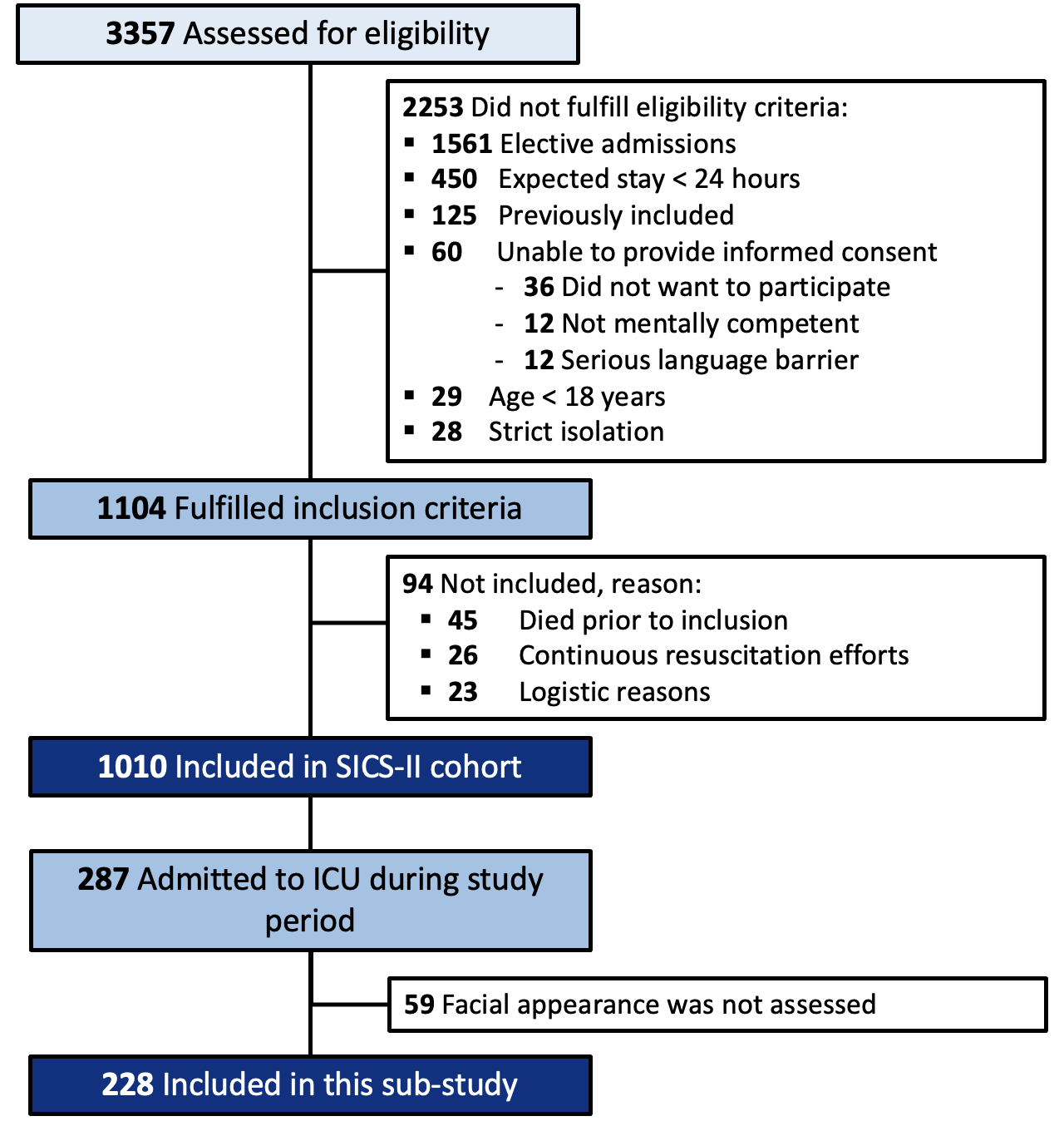
**

**Supplemental Figure 3.** Interaction between the two traits of gestalt; eye-opening and facial skin colour


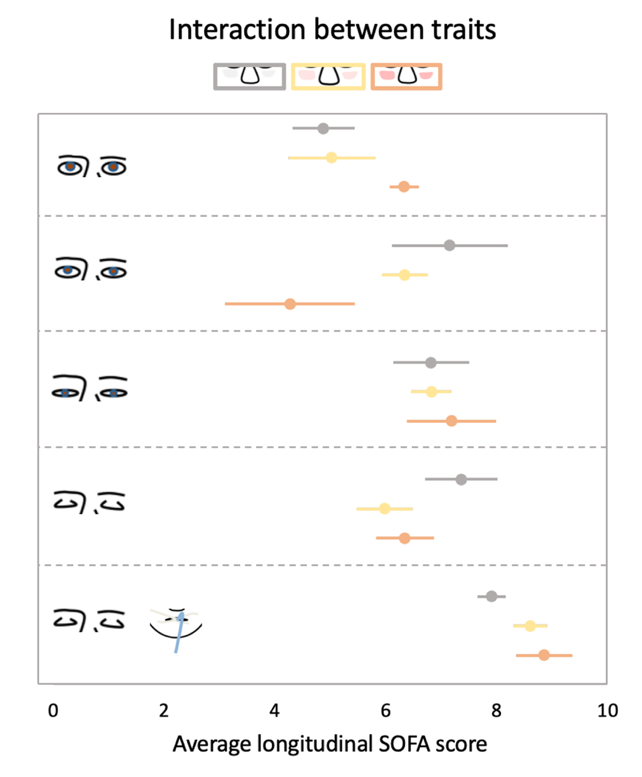


SOFA: Sequential Organ Failure Assessment; bars indicate standard error of the mean.

**Supplemental Table 1.** The SOFA score*^1^

|  | 1 | 2 | 3 | 4 |
| --- | --- | --- | --- | --- |
| Respiration  PaO2/FiO2 (mmHg) | ≤400 | ≤300 | ≤200^a^ | ≤100^a^ |
| Coagulation  Platelets (x10^9^ /l) | ≤150 | ≤100 | ≤50 | ≤20 |
| Liver  Bilirubin (μmol/l) | 20-32 | 33-101 | 102-204 | >204 |
| Cardiovascular  Hypotension^b^ | MAP <70 mmHg | Dopamine ≤5 or dobutamine (any dose) | Dopamine >5 or epinephrine ≤0.1 or norepinephrine ≤0.1 | Dopamine >15 or epinephrine >0.1 or norepinephrine >0.1 |
| CNS  Glasgow Coma Scale | 13-14 | 10-12 | 6-9 | <6 |
| Renal  Creatinine (μmol/l) or Urine output (ml/day) | 110-170 | 171-299 | 300-440 or  urine output <500 | >440 or  urine output <200 |

a: with respiratory support; b: adrenergic agents administered for at least one hour (doses given are in µg/kg × min).

Abbreviations: MAP: mean arterial pressure; CNS: central nervous system; SOFA: Sequential Organ Failure Assessment

*After completing the data, every patient had a SOFA score for each day at the ICU, resulting in 852 serial SOFA scores. On day one of admission, all patients had a SOFA score; on day two, 214 (94%) patients had a SOFA score, and on day three, 139 (61%) patients, and on day four, 96 (42%) patients had a SOFA score. On day five, 72 (32%) patients, day six, 58 (25%), and day seven, 45 (20%) patients had a SOFA score.

**Reference**

1. Vincent JL, Moreno R, Takala J, et al.: The SOFA (Sepsis-related Organ Failure Assessment) score to describe organ dysfunction/failure. On behalf of the Working Group on Sepsis-Related Problems of the European Society of Intensive Care Medicine. *Intensive Care Med* 1996; 22:707–10
